# The favorable kinetics and balance of nebivolol-stimulated nitric oxide and peroxynitrite release in human endothelial cells

**DOI:** 10.1186/2050-6511-14-48

**Published:** 2013-09-28

**Authors:** R Preston Mason, Robert F Jacob, J Jose Corbalan, Damian Szczesny, Kinga Matysiak, Tadeusz Malinski

**Affiliations:** 1 Cardiovascular Division, Department of Medicine, Brigham and Women’s Hospital, Harvard Medical School, 02115 Boston, MA, USA; 2 Elucida Research LLC, 01915 Beverly, MA, USA; 3 Department of Chemistry and Biochemistry, Ohio University, 45701 Athens, OH, USA

**Keywords:** Nevibolol, Nitric oxide, Peroxynitrite, ATP, β_3_-adrenergic receptors, P2Y-purinergic receptors

## Abstract

**Background:**

Nebivolol is a third-generation beta-blocker used to treat hypertension. The vasodilation properties of nebivolol have been attributed to nitric oxide (NO) release. However, the kinetics and mechanism of nebivolol-stimulated bioavailable NO are not fully understood.

**Methods:**

Using amperometric NO and peroxynitrite (ONOO^-^) nanosensors, β_3_-receptor (agonist: L-755,507; antagonists: SR59230A and L-748,337), ATP efflux (the mechanosensitive ATP channel blocker, gadolinium) and P2Y-receptor (agonists: ATP and 2-MeSATP; antagonist: suramin) modulators, superoxide dismutase and a NADPH oxidase inhibitor (VAS2870), we evaluated the kinetics and balance of NO and ONOO^-^ stimulated by nebivolol in human umbilical vein endothelial cells (HUVECs). NO and ONOO^-^ were measured with nanosensors (diameter ~ 300 nm) placed 5 ± 2 μm from the cell membrane and ATP levels were determined with a bioluminescent method. The kinetics and balance of nebivolol-stimulated NO and ONOO^-^ were compared with those of ATP, 2-MeSATP, and L-755,507.

**Results:**

Nebivolol stimulates endothelial NO release through β_3_-receptor and ATP-dependent, P2Y-receptor activation with relatively slow kinetics (75 ± 5 nM/s) as compared to the kinetics of ATP (194 ± 10 nM/s), L-755,507 (108 ± 6 nM/s), and 2-MeSATP (105 ± 5 nM/s). The balance between cytoprotective NO and cytotoxic ONOO^-^ was expressed as the ratio of [NO]/[ONOO^-^] concentrations. This ratio for nebivolol was 1.80 ± 0.10 and significantly higher than that for ATP (0.80 ± 0.08), L-755,507 (1.08 ± 0.08), and 2-MeSATP (1.09 ± 0.09). Nebivolol induced ATP release in a concentration-dependent manner.

**Conclusion:**

The two major pathways (ATP efflux/P2Y receptors and β_3_ receptors) and several steps of nebivolol-induced NO and ONOO^-^ stimulation are mainly responsible for the slow kinetics of NO release and low ONOO^-^. The net effect of this slow kinetics of NO is reflected by a favorable high ratio of [NO]/[ONOO^-^] which may explain the beneficial effects of nebivolol in the treatment of endothelial dysfunction, hypertension, heart failure, and angiogenesis.

## Background

Arterial endothelial cells modulate vascular tone through release of nitric oxide (NO), a potent vasodilator that regulates regional blood flow [1,2]. Beyond vasodilation, NO has various vascular benefits that reduce the risk for cardiovascular disease. NO inhibits smooth muscle cell proliferation and migration, adhesion of leukocytes to the vascular endothelium, and platelet aggregation [3]. An uncoupling of endothelial nitric oxide synthase (eNOS) along with reduced endothelial-dependent NO release and generation of peroxynitrite (ONOO^-^) has been linked to atherogenesis and its clinical manifestations [4,5]. Agents that enhance NO bioavailability have been shown to reduce cardiovascular events, as well as central arterial blood pressure, in patients with hypertension [4,5]. NO generation in the endothelium is accompanied by the production of ONOO^-^. Peroxynitrite, a major component of nitroxidative stress, is cytotoxic and can trigger a cascade of events leading to vasoconstriction, dysfunction of the endothelium, and apoptosis [6]. Therefore, a change in the balance between NO and ONOO^-^ generated by the endothelium can significantly affect the endothelial function, and as a result, lead to the dysfunction of the cardiovascular system.

ATP, which widely regulates cell and tissue function through autocrine or paracrine stimulation of purinergic (P2Y) receptors, has also been shown to be an important mediator of endothelial-dependent NO [7]. The vascular effect of ATP was first characterized in aortic segments from spontaneously hypertensive rats, as well as normotensive Wistar-Kyoto rats, in which direct application of ATP caused NO-mediated relaxation [8]. Similar effects were observed in hepatic arterial tissue isolated from New Zealand White rabbits and shown to be dependent on endothelial P2Y receptors [9]. In renal tissue, isolated from Wistar-Kyoto rats, ATP was further shown to induce relaxation of the glomerular microvasculature by activating P2Y receptors, followed by eNOS and guanylate cyclase pathway activation [10].

Nebivolol is a third-generation, β_1_-adrenergic receptor antagonist with vasodilatory properties that appear to be independent of its β_1_-receptor interactions [11-13]. Its mechanism of action is attributed to eNOS activation since its vasodilatory effects can be reversed with specific eNOS inhibitors such as N^G^-monomethyl-l-arginine (l-NMMA) and N_ω_-nitro-l-arginine methyl ester (l-NAME) [14-16]. In a number of independent studies, nebivolol-induced NO release has also been linked to β_3_-receptor interactions as well as ATP-dependent, P2Y-mediated eNOS activation [17-20]. Nebivolol has also been reported to reverse eNOS uncoupling and interfere with oxidative stress processes, by reducing NADPH oxidase activity or by directly scavenging oxygen-derived free radicals [13,20-23].

We conducted this study to evaluate simultaneously the kinetics of nebivolol-stimulated NO and ONOO^-^ production and the role of ATP efflux along with P2Y- and β_3_-receptor activation in human endothelial cells. We hypothesized that the slow kinetics of NO release in the endothelium, through integrated cellular mechanism that include both the ATP autocrine and/or paracrine pathway and these specific receptors, may be at least partially responsible for favorable balance between bioavailable NO and cytotoxic ONOO^-^. The high level of NO and low ONOO^-^ generated by nebivolol may explain its pleiotropic and therapeutic effects on the restoration of endothelial function in the cardiovascular system.

## Methods

### Materials

Nebivolol HCl (in powder form) was provided by Forest Research Institute (Commack, NY). The β_3_-agonist, L-755,507, and β_3_-antagonists, SR59230A and L-748,337, were purchased from Tocris Bioscience (Ellisville, MO). ATP, 2-MeSATP, and the non-selective P2Y receptor antagonist, suramin, were purchased from Sigma-Aldrich (St. Louis, MO). Gadolinium (Gd^3+^), a mechanosensitive, ATP-release channel blocker, superoxide dismutase (PEG-SOD) and the NADPH oxidase inhibitor, VAS2870, were also purchased from Sigma-Aldrich.

### Cell culture

Primary human umbilical vein endothelial cells (HUVECs) were purchased from Lonza Inc. (Walkersville, MD). Cells were cultured in the recommended complete endothelial cell growth medium and maintained at 37°C in a 95% air / 5% CO_2_ humidified incubator. As recommended by the supplier, cells were supplied with fresh medium every other day and propagated by an enzymatic (trypsin) procedure for a maximum of 16 population doublings. Our studies were performed in accordance with the guidelines established by the Ohio University Office of Institutional Research Compliance. These guidelines conform with the principles of the World Medical Association Declaration of Helsinki.

### NO and ONOO^-^ measurement

Endothelial NO and ONOO^-^ release was measured using amperometric nanosensors as previously described [21,24]. Briefly, each of the sensors was made by depositing a sensing material on the tip of a carbon fiber (length 4-5 μm; diameter 200-300 nm), i.e., a conductive film of polymeric nickel(II)tetrakis(3-methoxy-4-hydroxyphenyl)porphyrin for the NO sensor and a conductive film of polymeric manganese(III)-[2]paracyclophenylporphyrin for the ONOO^-^ sensor. The fiber was sealed with a nonconductive epoxy and connected to copper electrical wires with a conductive silver epoxy. Confluent HUVECs were rinsed with endothelial basal medium (EBM; Lonza Inc., Walkersville, MD) and the tandem of nanosensors was gently lowered to within 5 ± 2 μm from the surface of an endothelial cell using a remote-controlled micromanipulator (Sensapex, Finland). Amperometric measurements were performed using a Gamry Reference 600™ dual potentiostat (Gamry instruments, Warminster, PA). Basal NO and ONOO^-^ levels were measured by differential pulse voltammetry (DPV) in separate experiments. The DPV current at the peak potential characteristic for NO and ONOO^-^ is directly proportional to the local concentration of NO and ONOO^-^ in the immediate vicinity of the sensor. The nanosensors were calibrated before measurements in cells using a linear calibration curve (current versus concentration) constructed from standard NO or ONOO^-^ solutions ranging from 50 nM to 700 nM. The sensors response and calibration was tested again after measurements in cells, using the standard addition method. The detection limit of the sensors was 10^-9^ M.

Changes in current, proportional to the concentration of NO or ONOO^-^, were observed after the injection of nebivolol and other agents used in this study, including modulators of the ATP pathway and both agonists and antagonists of the β_3_-receptor. To test their direct effects, the compounds were administered acutely by a nanoinjector prior to measurements of NO and ONOO^-^ release from the cells. For combination studies, cells were treated with various β_3_-receptor and ATP pathway modulators, VAS2870 or PEG-SOD for 30 minutes prior to treatment with nebivolol.

### ATP measurement

Extracellular ATP was quantified using a luciferin-luciferase assay kit (BioAssay Systems, Hayward, CA). The ATP measurement was performed following the supplier’s recommendations. Briefly, confluent HUVECs were rinsed and incubated at 37°C in EBM medium for 5 minutes in the absence or presence of the various test agents. Aliquots (100 μL) of each sample supernatant were then transferred to a white opaque 96-well plate, along with luciferin and luciferase, and then luminescence was measured on a luminometer (BioTek, Winooski, VT). The ATP concentration was obtained using a standard calibration, prepared as recommended in the kit.

### Calculations and statistical analysis

All data are presented as mean ± standard deviation (SD) of the mean of n > 3. Statistical analysis of the mean difference between multiple groups was performed using one-way analysis of variance (ANOVA) with Student-Newman-Keuls multiple comparisons post hoc analysis; and between two groups, using Student’s *t*-test. The alpha level for all the tests was 0.05. A P value <0.05 was considered to be statistically significant. All statistical analyses were performed using Origin (v 6.1 for Windows; OriginLab, Northampton, MA) and GraphPad Prism (v. 5.00 for Windows; GraphPad Software, San Diego, CA).

## Results

**Figure 1:**
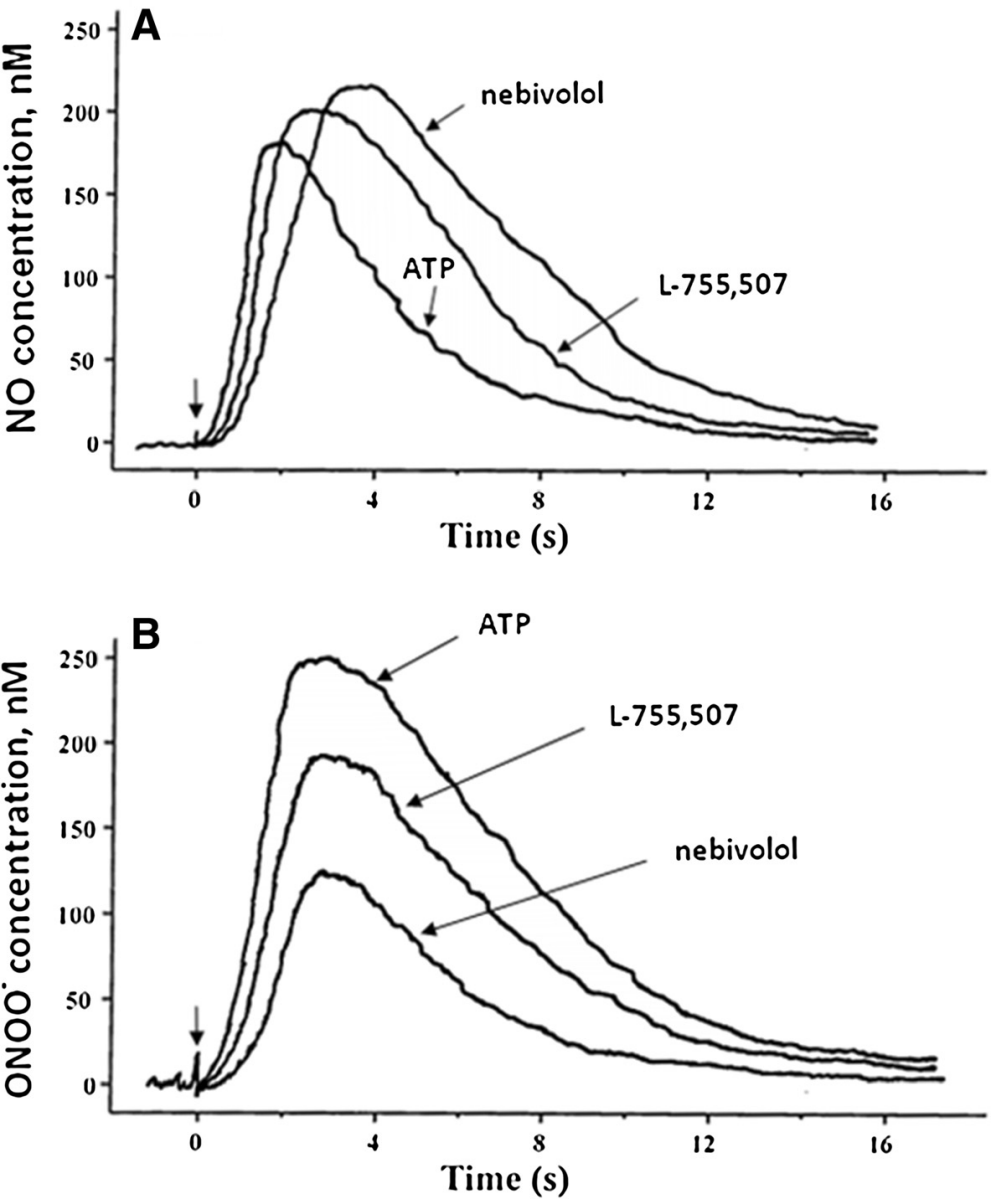
**Representative amperograms showing endothelial NO and ONOO**^**- **^**release stimulated with nebivolol, L-755,507, 2-MeSATP, and ATP.** NO **(A)** and ONOO^-^**(B)** release from HUVECs were stimulated with nebivolol, ATP, 2-MeSATP, or L-755,507 (each at 1 μM). The amperograms of the 2-MeSATP partially overlaps that of L-755,507 and are omitted. NO and ONOO^-^ release were measured with electrochemical nanosensors positioned 5 ± 2 μm from the surface of a single cell. Arrows indicate compound administration.

**Figure 2:**
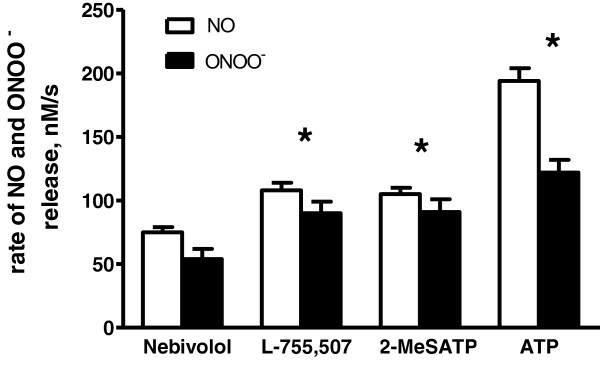
**Rate of endothelial NO and ONOO**^**- **^**release.** NO and ONOO^-^ release from HUVECs were stimulated with nebivolol, L-755,507, 2-MeSATP, and ATP (each at 1 μM). Values are mean ± SD (n = 5). One-way ANOVA, Student-Newman-Keuls multiple comparison post-hoc test: *P < 0.05 compared with either the rate of NO or ONOO^-^ released by cells stimulated with nebivolol.

Using nanonsensor technology, we measured *in situ*, near-real time NO and ONOO^-^ released from HUVECs following the acute administration of nebivolol, L-755,507, 2-MeSATP or ATP over a range of concentrations. Representative amperograms (concentration/current vs. time) collected from endothelial cells treated with nebivolol, L-755,507, 2-MeSATP, and ATP are shown in the Figure 1. A distinctive difference between the slope and peak height of amperograms was observed for both NO and ONOO^-^ production. The slope of amperograms was used to calculate the rate of NO and ONOO^-^ generation by endothelial cells after stimulation with nebivolol, L-755,507, 2-MeSATP, and ATP (Figure 2). The kinetics of NO release was relatively slow for nebivolol, with a rate of 75 ± 4 nM/s, and significantly faster for L-755,507 (108 ± 6 nM/s) and 2-MeSATP (105 ± 5 nM/s); and very fast for ATP-stimulated NO release (194 ± 10 nM/s). The rates for ONOO^-^ followed this same pattern as NO – lowest rate for nebivolol and the highest for ATP.

**Figure 3:**
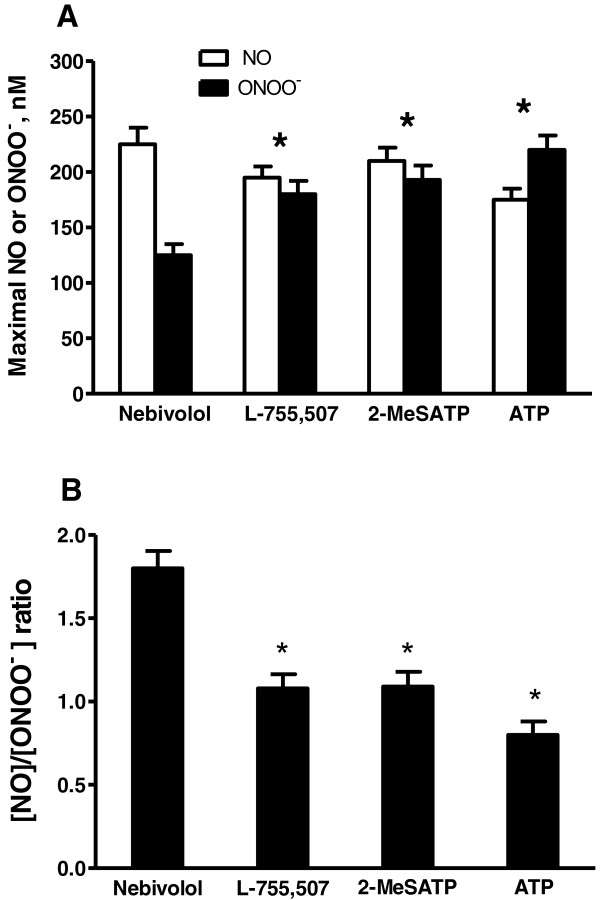
**Maximal endothelial NO and ONOO**^**- **^**concentrations, and [NO]/[ONOO**^**-**^**] ratio stimulated with nebivolol, L-755,507, 2-MeSATP, and ATP. (A)** Maximal NO and ONOO^-^ concentration release from HUVECs stimulated with nebivolol, L-755,507, 2-MeSATP, and ATP (each at 1 μM). Values are mean ± SD (n = 5). One-way ANOVA, Student-Newman-Keuls multiple comparison post-hoc test: *P < 0.05 compared with either the maximal NO or ONOO^**-**^ concentration released by cells stimulated with nebivolol. **(B)** The [NO]/[ONOO^-^] ratio calculated from the maximal concentration in A. Values are mean ± SD (n = 5). One-way ANOVA, Student-Newman-Keuls multiple comparison post-hoc test: *P < 0.05 compared with [NO]/[ONOO^-^] ratio calculated for nebivolol.

As shown in the Figure 3, the maximal NO concentration of 225 ± 15 nM was observed after stimulation with nebivolol and was the highest among the four agents tested. Surprisingly, ONOO^-^ concentration was the lowest after nebivolol stimulation (125 ± 10 nM) and the highest after ATP stimulation (220 ± 13 nM). The maximal NO and ONOO^-^ concentrations were between that observed for nebivolol and ATP. We applied the ratio of [NO] and [ONOO^-^] concentrations to depict the chemical redox balance between these two molecules in the cellular milieu. A decrease in [NO]/[ONOO^-^] ratio indicates a decrease in the concentration of the cytoprotective NO and/or an increase in the level of highly oxidative, cytotoxic ONOO^-^. A ratio of [NO]/[ONOO^-^] below 1.0 is an indicator that the cellular environment is dominated by high oxidative/nitroxidative stress.

Nanosensors provide unique opportunities for the simultaneous measurement of NO and ONOO^-^ concentration in small volume (~10^-15^ L), at near real-time (10^-5^ s) in close proximity to the cell membrane (~5 μm). The ratios of [NO]/[ONOO^-^] are presented in the Figure 3B. There is a highly significant difference in the [NO]/[ONOO^-^] balance between nebivolol (1.80 ± 0.10) and ATP (0.80 ± 0.08). The ratio of [NO]/[ONOO^-^] for L-755,507 and 2-MeSATP are similar, 1.08 ± 0.08 and 1.09 ± 0.09 respectively. There is a 40-60% difference in the [NO]/[ONOO^-^] balance between nebivolol and 3 other agents tested here.

**Figure 4:**
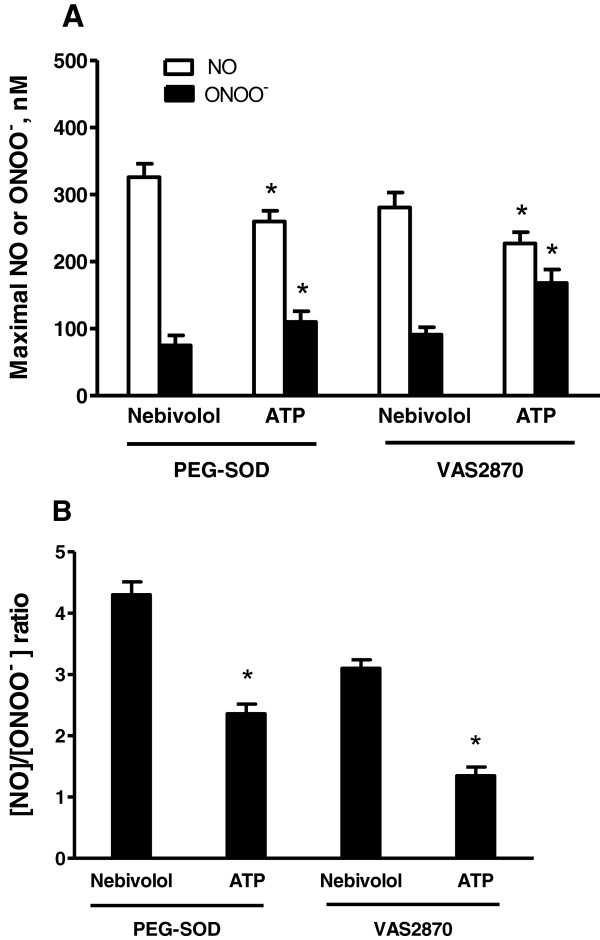
**Maximal endothelial NO and ONOO**^**- **^**concentrations, and [NO]/[ONOO**^**-**^**] ratio stimulated with nebivolol and ATP following a PEG-SOD and VAS2870 incubation. (A)** Maximal NO and ONOO^-^ concentrations stimulated with nebivolol and ATP (each at 1 μM) following a 30-minute incubation of HUVECs with PEG-SOD (400 U/mL) and VAS2870 (5 μM). Values are mean ± SD (n = 5). Student’s t-test: *P < 0.05 compared with nebivolol. **(B)** The [NO]/[ONOO^-^] ratio calculated from the maximal concentration in A. Values are mean ± SD (n = 5). Student’s t-test: *P < 0.05 compared with [NO]/[ONOO^-^] balance calculated for nebivolol.

A very low ratio of [NO]/[ONOO^-^] (lower than one) was observed only after the stimulation of endothelial cells by ATP (Figure 3B). We validated this model of monitoring [NO]/[ONOO^-^] balance in endothelial cells by changing the level of superoxide (O_2_^-^), the precursor of ONOO^-^. In the presence of membrane permeable PEG-SOD (400 U/mL), a significant reduction in ONOO^-^ concentration with concomitant increase in the NO level was observed (Figure 4A). This effect was observed for both nebivolol and ATP. A similar effect of the increase in NO and proportional decrease in ONOO^-^ was noticed in the presence of NADPH oxidase inhibitor, VAS2870 (5 μM). The Inhibition of the NADPH oxidase increases NO concentration by 20-30% after stimulation with nebivolol or ATP. This indicates that about 20-30% of NO produced by the endothelium is consumed by O_2_^-^ generated by NADPH oxidase. The source of the remaining 70-80% of O_2_^-^ in nebivolol- or ATP-stimulated endothelium is most likely eNOS.

The decrease in O_2_^-^ had a significant influence on the level of bioavailable NO and the concentration of ONOO^-^, as reflected by a significant increase in [NO]/[ONOO^-^] ratio (Figure 4B). A favorable [NO]/[ONOO^-^] balance increased even further for nebivolol to 4.30 ± 0.21 in the presence of PEG-SOD. Also, in the presence of PEG-SOD, a favorable shift in the [NO]/[ONOO^-^] balance was observed for ATP.

**Figure 5:**
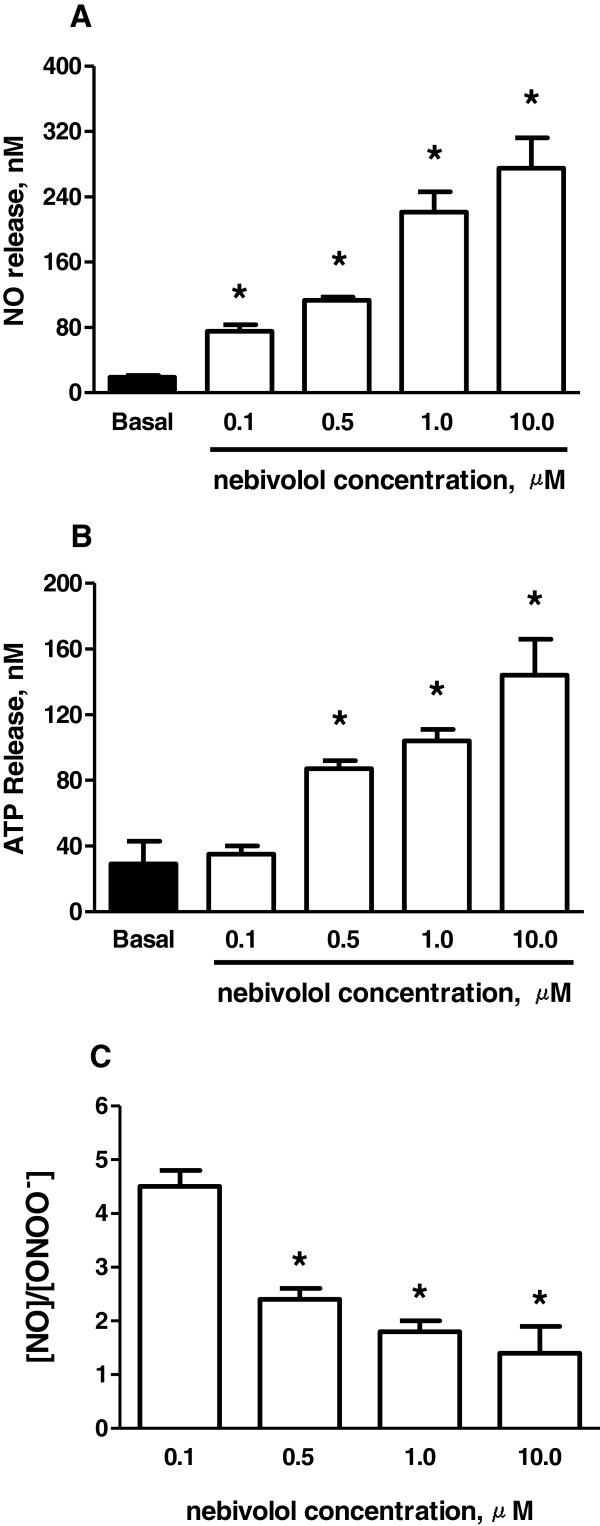
**Maximal endothelial NO concentration, ATP release, and [NO]/[ONOO**^**-**^**] ratio stimulated with nebivolol.** Maximal NO **(A)** and ATP **(B)** concentration release from HUVECs stimulated with different concentrations of nebivolol, along with the [NO]/[ONOO^-^] ratio **(C)**. Values are mean ± SD (n = 5). One-way ANOVA, Student-Newman-Keuls multiple comparison post-hoc test: *P < 0.05 compared with basal (in the absence of nebivolol) **(A, B)** and 0.1 μM nebivolol **(C)**.

Nebivolol increased endothelial NO release in a dose-dependent manner (Figure 5A). The effect of nebivolol on ATP concentration released from cells was significant and correlated well with a dose-dependent increase in NO production (Figure 5B). The ratio of [NO]/[ONOO^-^] decreased with the increase of nebivolol concentration (Figure 5C). This correlates well with a fast increase in nebivolol-stimulated ATP component in the overall stimulation process of NO release.

**Figure 6:**
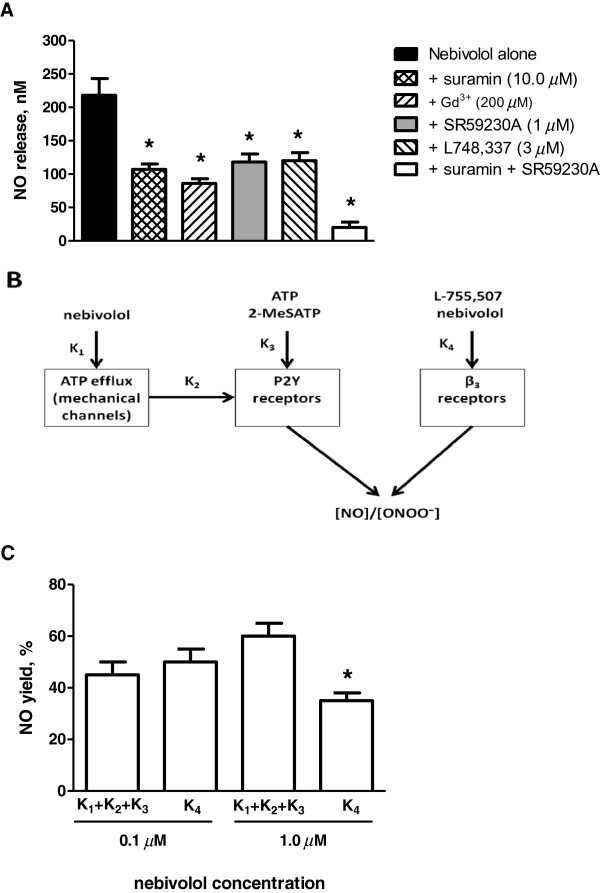
**Major pathways involved in nebivolol-stimulated NO and ONOO**^**- **^**generation, and maximal endothelial NO concentration stimulated with nebivolol alone or following an incubation with several modulators. (A)** Maximal NO concentration release from HUVECs stimulated with nebivolol (1 μM). Cells were also incubated with suramin (10 μM), Gd^3+^ (200 μM), SR59230A (1 μM), L-748,337 (3 μM) and suramin + SR59230A for 30 minutes and NO release was stimulated with nebivolol (1 μM). Values are mean ± SD (n = 5). One-way ANOVA, Student-Newman-Keuls multiple comparison post-hoc test: *P < 0.05 compared with nebivolol alone. **(B)** Nebivolol-stimulated generation of NO and ONOO^-^ involves at least two major pathways and several steps. **(C)** At low nebivolol concentration, K_4_ will be more determinant than at higher concentration. Values are mean ± SD (n = 5). Student’s t-test: *P < 0.05 compared with K_1_ + K_2_ + K_3_.

The relationship between NO bioavailability and ATP production was further tested using modulators of the ATP/purinergic pathway. Each of these agents significantly attenuated the effects of nebivolol on endothelial NO release (Figure 6A). At the specific concentrations tested, suramin (10 μM) and Gd^3+^ (200 μM) inhibited nebivolol-induced NO release by 50 and 60%, respectively. These findings are consistent with the observation that the effects of nebivolol on endothelial-dependent NO release is casually associated with ATP production, especially at higher concentrations of nebivolol. We also measured the effects of nebivolol on endothelial NO release in the presence of β_3_-receptor antagonists SR59230A (1 μM) or L-748,337 (3 μM). Both of these agents reduced the nebivolol-induced NO release by approximately 50% (Figure 6A). However, a combination of suramin and SR59230A reduced nebivolol stimulated NO by more than 90%.

## Discussion

The key finding from this study is that nebivolol-stimulated NO release from human endothelial cells is multipathway and slow. This slow process preserves eNOS coupling and leads to a high production of bioavailable NO and low production of ONOO^-^. The slow kinetics and dynamics of NO generation is a significant factor in the maintaining of the highly favorable balance between [NO] and [ONOO^-^] concentrations in the endothelium. The favorable kinetics of NO release, combined with O_2_^-^ scavenging by nebivolol, may help to explain the pleiotropic effect of nebivolol on the cardiovascular system observed in clinical studies. The rate of NO release by nebivolol is slower than that observed for the other three agents presented in this study (ATP, L-755,507, and 2-MeSATP). These three agents produced comparable NO concentrations with nebivolol, however, excessive and rapid NO production stimulated by these agents eventually leads to uncoupling of eNOS (rapid depletion of substrates and/or cofactors for NO production). The uncoupled eNOS is an efficient generator of O_2_^-^ in one electron transfer reduction of oxygen. Therefore, uncoupled eNOS can produce, sequentially, both NO and O_2_^-^. NO and O_2_^-^ generated in close proximity by eNOS can react rapidly in a diffusion controlled reaction to produce ONOO^-^. The studies with VAS2870 also elucidated that the second major source of O_2_^-^ in the endothelium during the stimulation of NO release by nebivolol is NADPH oxidase. Our study shows that after the stimulation of endothelial cells with nebivolol, the contribution of NADPH oxidase to the pool of O_2_^-^, and subsequently the pool of ONOO^-^ is about 20-30%, while about 70-80% of O_2_^-^ and ONOO^-^ comes from uncoupled eNOS. NADPH oxidase contribution to the pool of O_2_^-^ and ONOO^-^ after stimulation with ATP is about 30-35% with eNOS contributing 65-70%. In addition to the favorable kinetics of NO release, nebivolol may also increase NO bioavailability through non-receptor-mediated mechanisms, such as conveying antioxidant benefits of the endothelium. Nebivolol has been shown to scavenge O_2_^-^ independent of β_3_-receptor blockade in animal and cell based models of cardiovascular diseases [13,17,20,21]. These effects are attributed to its specific interactions with plasma membrane and its efficiency as a chain-breaking antioxidant [13,25]. Nebivolol has also been shown to interact with enzymatic sources of oxygen radicals such as NADPH oxidase [21,22]. This correlates well with our data showing lower generation of O_2_^-^/ONOO^-^ by NADPH oxidase than eNOS after stimulation with nebivolol.

The scavenging properties of nebivolol cannot alone explain the low level of ONOO^-^ and slow kinetics of nebivolol stimulated NO production. The results of our study suggest that nebivolol increases NO release in the human endothelium through a complementary mechanism involving β3-receptor, ATP autocrine and/or paracrine, and P2Y-receptor activation. Two different β_3_-receptor antagonists (SR59230A and L-748,337) were discovered to significantly reduce nebivolol-induced NO release in HUVECs. However, these β_3_-receptor antagonists reduced NO production only by about 50%. A blockage with Gd^3+^ of mechanosensitive ATP channels of HUVECs reduced NO production by 60%, indicating a direct involvement of extracellular ATP in the stimulation process. Finally, in the presence of both antagonists of the P2Y-receptor, suramin, and β_3_-receptor antagonist, SR59230A, NO concentration decreased by more than 90%.

Our findings in this study argue for the involvement of β_3_-receptors in eNOS activation and NO release in human endothelial cells stimulated by nebivolol. A role for β_3_-receptors in nebivolol-induced NO release was previously demonstrated in human heart ventricular tissue and coronary microarteries [19]. Nebivolol was shown to activate cardiac β_3_-receptors in a manner similar to that of the selective β_3_-receptor agonist, BRL 37344, both of which resulted in a change in ventricular contraction attributed to NO release. The negative inotropic effects of nebivolol were modified by pretreatment with L-748,337, but not with nadolol, a nonselective β_2_/β_3_-receptor antagonist [19]. These specific receptor-mediated effects of nebivolol on NO metabolism may contribute to favorable changes in vascular hemodynamic properties and calcium regulation given the relative distribution of β_1_- and β_3_-receptors in the failing heart. Clinical support for such potential benefit was demonstrated in a randomized trial of elderly patients with documented heart failure [26]. Nebivolol was also shown in another study to increase vasodilation in coronary microarteries essential for the regulation of coronary resistance and perfusion reserve [18]. Endothelial-dependent vasodilation was not reproduced with nebivolol in mice deficient for β_3_-receptors [18]. Nebivolol also failed to induce neocapillary tube formation in animals deficient in either β_3_-receptor or eNOS expression [18]. Another recent study showed that nebivolol increased levels of endothelial progenitor cells, promoted angiogenesis, and reversed left ventricular dysfunction in mice with extensive myocardial infarction [20]. The vasodilation effects of nebivolol could only be partially blocked with a specific β_3_-adrenergic receptor antagonist [20]. The data presented in our work established an important connection between the cardioprotective effects of nebivolol and its β_3_-mediated, ATP-mediated effects on eNOS function.

The results of this study also suggest a role for the ATP autocrine and/or paracrine pathway in the activation of eNOS. It was found that the mechanosensitive ATP channel blocker, Gd^3+^, inhibited nebivolol-induced NO release by 60%. Moreover, the rate of NO stimulation with ATP is much faster than the stimulation with nebivolol. Therefore, we concluded that a rate determining factor in the kinetics of nebivolol-stimulated NO may be the ATP efflux from endothelial cells. The delivery of ATP from intracellular to extracellular space will require a buildup of the gradient of concentration, passage through mechanical channels and diffusion to receptors on the membrane surface. These delivery processes, based on efflux and diffusion, will be much slower than the direct high gradient diffusion of ATP to membrane receptors from an outer solution of ATP.

Extracellular ATP promotes vascular relaxation through the activation of P2Y receptors and the subsequent stimulation of eNOS and cytosolic guanylate cyclase [10]. Exogenous ATP has been shown to promote NO release from Wister-Kyoto rat glomerular endothelial cells with kinetic properties similar to those of nebivolol [17]. Inhibition of ATP efflux with Gd^3+^, an inhibitor of stretch-activated channels, also reduced the effects of nebivolol [17]. We demonstrated that a pretreatment of cells with Gd^3+^ decreased the ability of nebivolol-induced endothelial NO release. This may suggest that nebivolol itself may be linked to opening of mechanosensitive ATP channels.

It appears from this study that the kinetics of NO production by eNOS is crucial in maintaining a favorable balance between [NO]/[ONOO^-^] concentrations. A rapid stimulation may produce high level of NO but also a high level of ONOO^-^. Therefore, the rapid generation of NO accompanied by high ONOO^-^ cancels the beneficial effect of NO and imposes a deleterious effect of ONOO^-^ -induced nitroxidative stress with severe side effect for the endothelium. NO and ONOO^-^ stimulation by cerivastatin is a good example of this kind of “non-favorable kinetics” of NO release [27]. A potentially excellent pleiotropic effect of cerivastatin was compromised by the negative effect of high ONOO^-^ generated by this drug. This negative side effect of cerivastatin on the cardiovascular system was the forced withdrawal of this otherwise excellent drug from the pharmaceutical market.

### Limitations

HUVECs were used in this study as the sole source of endothelial cells. Further studies will be required to confirm these findings using other sources of endothelial cells.

## Conclusions

We propose that nebivolol-stimulated generation of NO and ONOO^-^ involves at least two major pathways and several steps (Figure 6B). One of this pathways involves a stimulation of intracellular ATP efflux through mechanical channels (K_1_), diffusion of extracellular ATP to P2Y receptors (K_2_), and stimulation of P2Y receptors by ATP (K_3_) followed by the release of NO and ONOO^-^. We propose that this pathway involving many steps is a rate determining factor in NO and ONOO^-^ production after stimulation with nebivolol. The other pathway, through β_3_ receptors (K_4_) is faster and the rate of NO and ONOO^-^ release comparable with that of K_3_. Therefore, the yield of NO produced by each pathway will vary with the concentration of nebivolol. At low nebivolol concentration, K_4_ pathway will be more determinant than at higher concentration (Figure 6C).

The results of this study provide additional insights into the cellular basis for nebivolol-induced NO release in human endothelial cells. The ability of nebivolol to stimulate NO release appears to be independent of its selective β_1_-blockade properties and dependent on stimulation of β_3_-receptors and ATP-mediated stimulation of P2Y-purinergic receptors. It seems to be also linked to direct opening of mechanosensitive ATP channels. A multistep stimulation of NO release is relatively slow and the production of NO does not significantly influence the supply of substrates or cofactors to eNOS, maintaining its relative high degree of coupling. The coupled eNOS can produce high NO concentration and low ONOO^-^ leading to a highly beneficial effect of nebivolol in the treatment of dysfunctional endothelium in cardiovascular diseases.

## Competing interests

Dr Mason received an independent research grant in support of this study from Forest Laboratories. All other authors have no conflicts of interest to disclose.

## Authors’ contributions

RPM and TM designed the study. JJC, DS, and KM performed the experimental work. RPM, RFJ, JJC, and TM carried out the analyses, interpreted the data, drafted the manuscript and critically reviewed it. All authors read and approved the final manuscript.

## Pre-publication history

The pre-publication history for this paper can be accessed here:

http://www.biomedcentral.com/2050-6511/14/48/prepub
